# Improved Singlet Oxygen Production by Synergistic Effect *via* a Dual-Core Photosensitizer Doped Polymer Fibrous Films: Synthesis and Performance

**DOI:** 10.3389/fchem.2022.890545

**Published:** 2022-05-09

**Authors:** Ming Zhao, Cheng Liu, Zhihao Shan, Changqing Ji, Xiaozhong Lu, Guanglei Lv

**Affiliations:** ^1^ College of New Materials Science and Chemical Engineering, Beijing Institute of Petrochemical Technology, Beijing, China; ^2^ Institute of Environmental Protection Disposal Technology of Polymer Materials, Beijing Institute of Petrochemical Technology, Beijing, China; ^3^ Beijing Key Laboratory of Specialty Elastomer Composite Materials, Beijing Institute of Petrochemical Technology, Beijing, China; ^4^ CNOOC (Tianjin) Pipeline Engineering Technology Ltd., Tianjin, China

**Keywords:** photosensitizer, Re(I) complex, phosphorescent emission, singlet oxygen, synergistic effect

## Abstract

Singlet oxygen (^1^O_2_) is a common reactive oxygen species that has found wide application in wastewater processing, photochemical synthesis, and photodynamic therapy. In this paper, a dual-core metal [a Re(I)-based component and a Gd(III)-based component] photosensitizer was synthesized and doped into polymer fibrous films for ^1^O_2_ generation. Here the Re(I)-based component is responsible for the photosensitizing reaction which directly transformed ^3^O_2_ to ^1^O_2_, while the Gd(III)-based component served as an auxiliary part that assisted the transformation from ^3^O_2_ to ^1^O_2_ *via* synergistic effect by its triplet excited ligands. The photophysical parameters of this photosensitizer (denoted as Re-Gd) and its fibrous films (denoted as Re-Gd@PVP) were carefully recorded, discussed, and compared. It was found that the excited state lifetime and photostability of Re-Gd were both improved after being doped into fibrous films, favoring ^1^O_2_ generation. The ^1^O_2_ generation performance comparison between Re-Gd in the solid state, in solution, and fibrous films suggested that ^1^O_2_ generation performance was indeed improved by the electrospinning films. In addition, the positive factor of synergistic effect on improving ^1^O_2_-producing efficiency was confirmed by comparing Re-Gd@PVP films with reference films with a single-core metal photosensitizer having no synergistic effect.

## Introduction

Singlet oxygen (^1^O_2_) is a common reactive oxygen species (ROS) that has found wide applications in wastewater processing, photochemical synthesis, and photodynamic therapy (Ishi-i et al., 2007; Li et al., 2016). A direct transformation from triplet oxygen (^3^O_2_) to ^1^O_2_, however, is highly prohibited due to the spin rule. As a consequence, a photosensitizer is usually applied to assist such transformation. With the help of the photosensitizer upon suitable excitation wavelength, the photosensitizer is excited from its ground state (S_0_) to the first singlet excited state (S_1_) and then transformed into the first triplet excited state (T_1_) *via* intersystem crossing (ISC). A direct interaction/collision between photosensitizer T_1_ and ^3^O_2_ yields ^1^O_2_ (Hou et al., 2014; Liu et al., 2015).

Some commonly used photosensitizers include phthalocyanine derivatives, methylthionine chloride derivatives, porphyrin derivatives, and transition metal complexes (Yao et al., 2012). Among these candidate photosensitizers, pure organic ones have shown excellent ^1^O_2_-producing yield, but their limited photostability partially compromises their performance. In this case, luminescent transition metal complexes have been nominated as a photosensitizer owing to the participation of the spin-orbit coupling effect which facilitates the ISC transformation from S_1_ to T_1_, favoring ^1^O_2_ generation (Zhang et al., 2012). Various luminescent metal complexes have been tried, such as Cu(I), Re(I), Ru(II), Pt(II), and traditional rare-earth (RE) complexes (Cakmak et al., 2011; Lai et al., 2013; Ding and Han, 2015; Wang et al., 2015). Generally speaking, Re(I) complexes with diimine ligands have shown promising features such as high ^1^O_2_-producing yield, efficient absorption for excitation wavelength, long excited-state lifetime, and good photostability, which makes them a class of attractive photosensitizer. On the other hand, solid Re(I) photosensitizers suffer from negative effects of photobleaching, intense aggregation, and concentration quenching, decreasing ^1^O_2_-producing yield (Yogo et al., 2005).

To solve these problems, it has been proposed that Re(I) photosensitizer molecules should be dispersed in a supporting structure with many microspores, so that each Re(I) photosensitizer molecule is immobilized in a microspore, decreasing aggregation and concentration quenching effect. In addition, the supporting structure may offer a protective environment for Re(I) photosensitizer molecules, improving their photostability. There should be some criteria when choosing a supporting structure. For example, uniform microspores to ensure homogeneous photosensitizer distribution, large specific area to ensure enough adsorption capacity, and good compatibility with a photosensitizer to avoid phase separation. After listing the above criteria, it is found that fibrous polymer films prepared by electrospinning technology satisfy all of them (Huang et al., 2003; Wang et al., 2009). There have been some efforts about ^1^O_2_ production from electrospun fibers doped with photosensitizers (Huang et al., 2003; Wang et al., 2009). On the other hand, their performance is still yet to be satisfied. It is found that although their excited state lifetime has been prolonged after being doped into polymer fibers, their emission cannot be fully quenched, which means an incomplete energy transfer from photosensitizer T_1_ and ^3^O_2_. Some pieces of literature attribute such effect to the limitation of conjugation size in diimine ligand, but no matter how increased diimine ligand conjugation size is, photosensitizer emission is always observed (Knoll et al., 2015). As a consequence, alternative solutions should be figured out.

**SCHEME 1:**
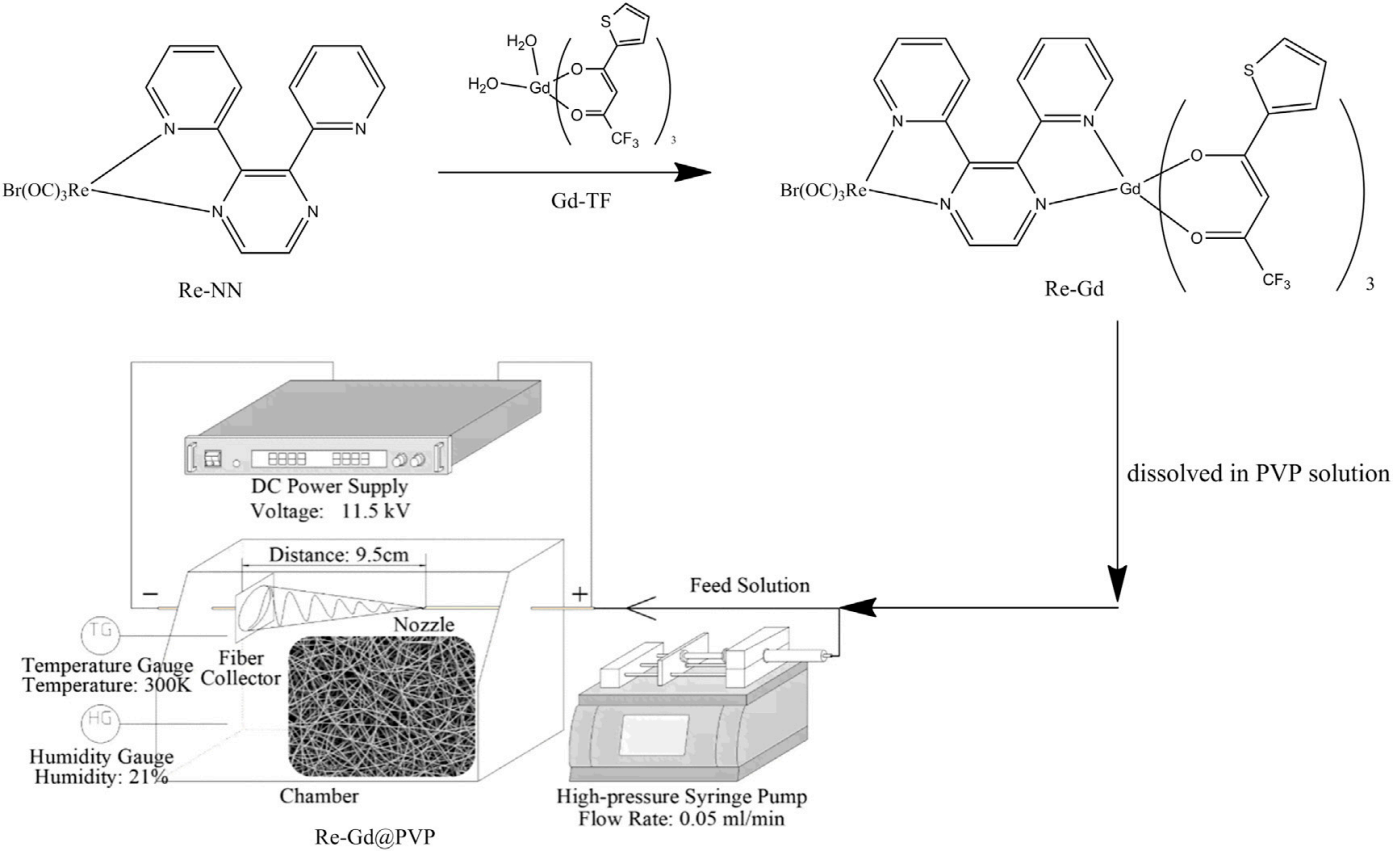
A schematic route for the synthesis of Re-Gd photosensitizer and Re-Gd@PVP fibers.

In this work, we intend to use a dual-core metal complex as the photosensitizer, one core is luminescent Re(I), and the other is nonluminescent Gd(III) coordinated with traditional β-deketone ligand, as shown in Scheme 1. Then this dual-core photosensitizer (denoted as Re-Gd) is doped into PVP (polyvinyl pyrrolidone) fibers *via* the electrospinning method (denoted as Re-Gd@PVP) to evaluate their ^1^O_2_-producing performance, where PVP had been proved a promising host for electrospinning due to its virtues of mechanical strength, O_2_ penetration, and stable photostability (Wang et al., 2009). It is expected that the synergistic effect between these two metal cores shall improve the energy transfer from photosensitizer T_1_ and ^3^O_2_ and thus increases ^1^O_2_-producing yield.

## Experiment Details

### Materials and Equipment

A schematic route for the synthesis of Re-Gd photosensitizer and Re-Gd@PVP fibers is shown in Scheme 1. The starting compounds of Re-NN and Gd-TF were synthesized following a literature procedure (Kennedy et al., 2007). PVP (K30), Re(CO)_5_, GdCl_3_•6H_2_O, 4,4,4-trifluoro-1-(thiophene-2-yl)butane-1,3-dione, NaOH, and related reagents were commercially obtained. Organic solvents were AR-grade ones and purified before usage. The NMR analysis was done using a Varian INOVA 300 spectrometer. The MS analysis was done using an Agilent 7500 ICP-MS spectrometer. Photophysical spectra and photodynamics were monitored using a Shimadzu spectrometer (Model 3100), a Hitachi F−7000 spectrometer, a Hitachi S-4800 microscope, and a Nikon TE2000-U fluorescence microscope.

### Synthesis of Re-Gd

With the previously synthesized Re-NN and Gd-TF as starting compounds, Re-Gd was synthesized as follows: a mixture of Re-NN and Gd-TF (2 mmol:2 mmol) was poured into dehydrated toluene (25 ml); at 115°C, this solution was heated and stirred for a whole day; then solvent was extracted by rotary evaporation; solid residue was purified on a silica gel column; the eluent was hexane and CH_2_Cl_2_ (v:v = 30:1); yellow powder was obtained as Re-Gd. ^1^H NMR (400 MHz, DMSO-*d*_6_): *δ* 8.74 (2H, s), 8.65 (2H, s), 8.33–8.29 (2H, m), 7.78–7.75 (2H, m), 7.47 (3H, m), 7.33–7.30 (m, 5H), 7.14–7.10 (m, 3H), 6.61 (s, 3H). ^13^C NMR: *δ* 183.99, 155.78, 154.50, 150.41, 150.11, 148.91, 148.65, 147.50, 147.03, 143.25, 143.01, 140.47, 132.88, 132.39, 132.32, 128.09, 123.13, 117.38, 82.34, 82.25, 82.15. MS Calcd. For C_41_H_22_BrF_9_GdN_4_O_9_ReS_3_: 1406.84. Found: [m-H]^+^ 1405.8. Elemental analysis for C_41_H_22_BrF_9_GdN_4_O_9_ReS_3_: C, 35.04; H, 1.58; N, 3.99. Found: C, 34.93; H, 1.65; N, 3.86.

### Fabrication of Re-Gd@PVP Fibers

The Re-Gd@PVP fibers were fabricated by the electrospinning method. A feed solution was first prepared as follows: solid PVP and Re-Gd were weighed and mixed with DMF solution; this solution was stirred gently to get a transparent solution (20 wt%); then this feed solution was connected to a high-pressure syringe pump which was wired to the positive terminal of a DC power supply (11.5 kV); a piece of Al foil was connected to the negative terminal of the above DC power supply and used as a fiber collector; the flow rate of the high-pressure syringe pump was set as 0.05 ml/min, the collector-nozzle distance was 9.5 cm, the temperature was 300 K, and humidity was 21%.

A reference sample Re-NN@PVP was prepared following the same protocol with a doping concentration of 5 wt%.

### Monitoring of ^1^O_2_ Production

To compare the ^1^O_2_-producing performance of Re-Gd@PVP fibers, each solid product was filled in a quartz flask with two tubes. Upon constant excitation on this solid production, the O_2_ stream was pumped into this quartz flask (10 ml/min) and then bubbled DPBF solution. Here DPBF (1,3-diphenylisobenzofuran) was dissolved in ethanol (1 mM) to serve as a ^1^O_2_ tracer. The DPBF solution was monitored by using an absorption spectrometer with an interval time of 2 min.

## Results and Discussion

### Molecular Structure Design of Re-Gd Photosensitizer

For a visual understanding on the molecular structure of Re-Gd, its geometry is simulated and shown in Supplementary Figure S1. As shown in Scheme 1, Re-Gd is a photosensitizer with two metal cores, a Re(I)-based one and a Gd(III)-based one. The former one is emissive and clearly designed to directly assist the interaction/collision between Re(I) T_1_ and ^3^O_2_ which yields ^1^O_2_. While the latter is nonemissive and shall not be directly useful for the direct production of ^1^O_2_. On the other hand, there is also a spin-orbit coupling effect in Gd(III)-based component. This spin-orbit coupling effect of Gd(III) makes its ligands take their T_1_ state after being excited. There may be even energy transfer and roll-back between Re(I) T_1_ and Gd(III)-ligand T_1_, which actually increases the excited state lifetime of T_1_ and thus the collision probability between T_1_ and ^3^O_2_. As a consequence, the synergistic effect in Re-Gd shall improve ^1^O_2_-producing yield.

### Photophysical Parameters of Re-Gd Photosensitizer

**FIGURE 1:**
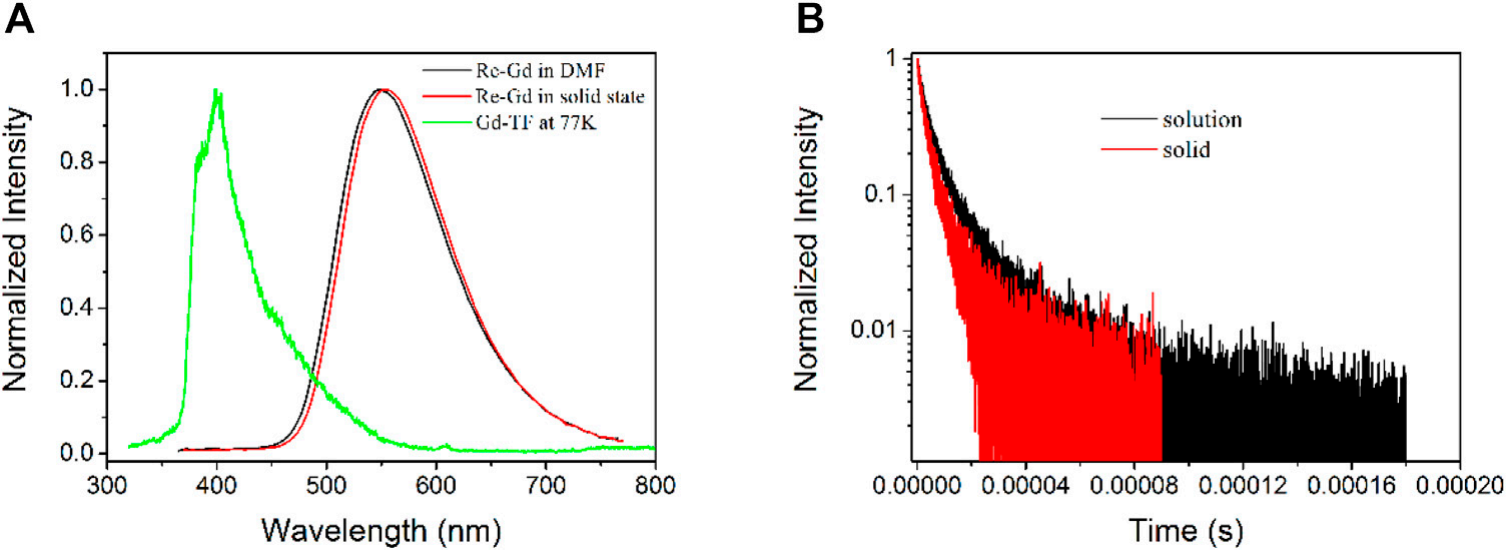
**(A)** Emission spectra of Re-Gd in solid state and DMF solution and that of Gd-TF at 77 K. **(B)** Emission decay dynamics of Re-Gd in solid state and DMF solution.

For a tentative confirmation of this hypothesis, the triplet state of Gd-TF is first determined by its phosphorescence spectrum at 77 K. The emission spectrum of Re-Gd is shown in Figure 1 for comparison. It is observed from Figure 1 that Re-Gd shows a standard Gaussian-shaped emission band, peaking at 548 nm in solid state and 556 nm in DMF solution. Its phosphorescent nature is confirmed by the emission decay dynamics shown in Figure 1. It is clear that Re-Gd follows a biexponential decay pattern, with a lifetime of 4.9 μs in solution and 3.6 μs in the solid state. These microsecond-scaled lifetimes confirm the phosphorescent nature of Re-Gd emission. In addition, a slight emission redshift is observed for Re-Gd in the solid state, compared to that in solution. This observation is explained by the aggregation-induced quenching effect. This explanation is consistent with the decreased lifetime in the solid state compared to that in the solution. On the other hand, the observation of such a biexponential decay pattern suggests that there is still a short-lived emissive component in the excited state which was not efficient and applicable in producing ^1^O_2_.

According to the literature reports, the emission of phosphorescent Re(I) complexes comes from their excited MLCT state (Yaghi et al., 2003). Here MLCT means metal-to-ligand-charge-transfer. The excited electrons are located at diimine ligands and their π* orbitals, corresponding to the first triplet state T_1_. Thus, the T_1_ level of the Re(I) component in Re-Gd shall be determined as 2.23 eV (556 nm) based on the aforementioned emission spectra. This value is much lower than the T_1_ level of Gd-TF determined by its low temperature (77 K) phosphorescence spectrum shown in Figure 1. As a consequence, the Gd(III) component in Re-Gd may serve as a secondary T_1_ energy donor for the Re(I) component, so that the ^1^O_2_-producing yield can be improved.

### Micromorphology Analysis of Re-Gd@PVP Fibers

**FIGURE 2:**
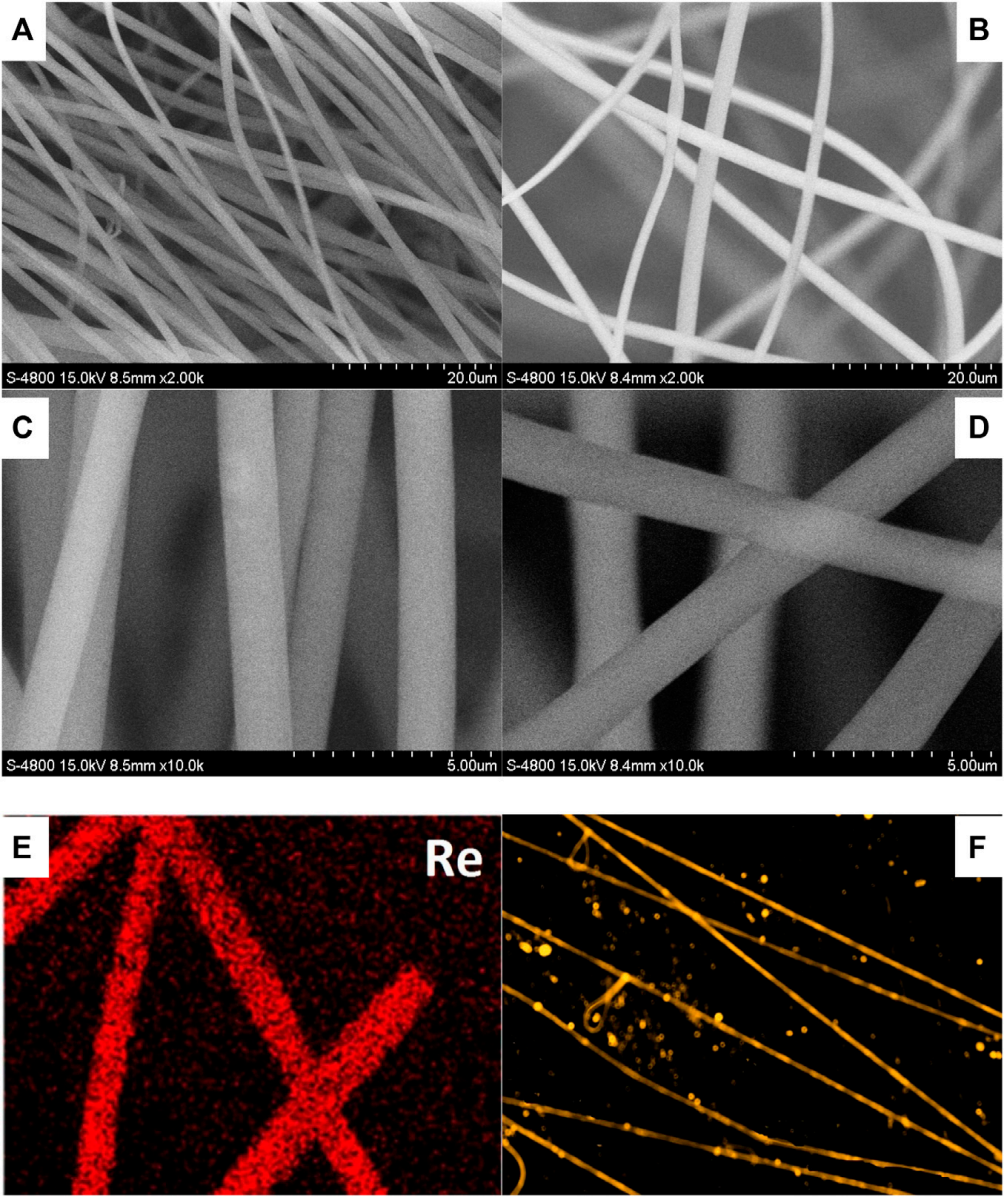
SEM images of Re-Gd@PVP [3 wt%, **(A,C)**; 5 wt%, **(B,D)**], Re elemental distribution map of Re-Gd@PVP [5 wt%, **(E)**] and fluorescence microscopy image of Re-Gd@PVP [5 wt%, **(F)**].

Due to the multiple rigid pyridine rings in the diimine ligand, Re-Gd suffers from a limited solubility in organic solvents such as CH_2_Cl_2_ and acetone. To ensure a fluent electrospinning procedure, DMF was used as a feed solution. A doping level of 5 wt% (vs. host weight) was applied in Re-Gd@PVP fibers (denoted as 5 wt% Re-Gd@PVP). An even higher doping level tended to cause aggregation and phase separation in the resulting electrospinning fibers. For comparison, a lower doping level of 3 wt% was applied to fabricate the Re-Gd@PVP fibers as well (denoted as 3 wt% Re-Gd@PVP). Their micromorphology is firstly discussed by their SEM images shown in Figure 2. Uniform long fibers are randomly distributed on the substrate. A dense but porous structure is formed by these fibers crossing each other. A smooth surface is observed, with no knots or junctions. Their mean diameters are determined as 1.5 μm for 3 wt% Re-Gd@PVP and 2.0 μm for 5 wt% Re-Gd@PVP, respectively. The increased doping level shall increase the viscosity of the feed solution, leading to the thicker 5 wt% Re-Gd@PVP fibers. The uniform dopant distribution of Re-Gd in 5 wt% Re-Gd@PVP is confirmed by its Re elemental distribution map shown in Figure 2. No sign of phase separation or aggregation is observed. Uniform yellow emission is observed from these Re-Gd@PVP fibers. Thus, it is confirmed that Re-Gd molecules have been uniformly dispersed in PVP fibers. There are emissive spots in Figure 2F, though. Considering the uniform Re distribution, they are considered fragments of electrospun fibers.

### Photophysical Parameters of Re-Gd@PVP Fibers

**FIGURE 3:**
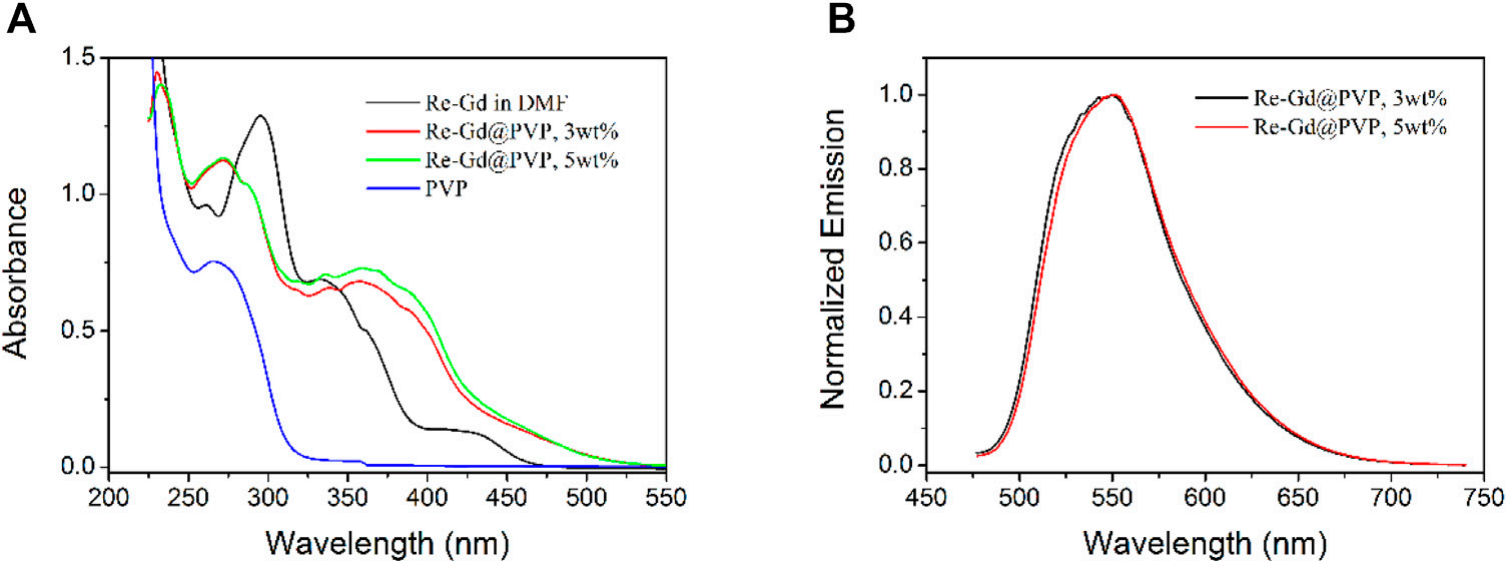
**(A)** Absorption spectra of Re-Gd (in DMF), PVP, and Re-Gd@PVP. **(B)** Emission spectra of Re-Gd@PVP.

The absorption spectra of Re-Gd@PVP fibers (3 wt% and 5 wt%), along with those of Re-Gd and PVP, are shown in Figure 3. As for Re-Gd, there are three absorption bands, a sharp one peaking at 296 nm, a broad one around 339 nm, and a weak and broad one at 430 nm. According to the literature reports, the last one (430 nm) shall be attributed to MLCT absorption from Re(I)-based component, while the second one (339 nm) shall be n-π* transitions in TF ligand of Gd(III)-based component. The first ligand shall be the absorption combination of Re(I)-based and Gd(III)-based components (Paw and Eisenberg, 1997; Yaghi et al., 2003). The absorption spectra of Re-Gd@PVP fibers (3 wt% and 5 wt%) are basically the absorption combination of Re-Gd and PVP, showing absorption peaks of 272 nm (with a shoulder peak of 289 nm), 367, and 450 nm. The first absorption peak is attributed to PVP host absorption, and its shoulder peak is attributed to that originated from Re-Gd. The second one (367 nm) and the third one (450 nm) are assigned to Re-Gd absorption, which corresponds to n-π* transitions in the TF ligand of Gd(III)-based component and MLCT absorption from Re(I)-based component, respectively. A redshift is clearly observed for these Re-Gd@PVP absorption peaks, compared to corresponding absorption peaks of Re-Gd. This is attributed to the solid aggregation effect since Re-Gd molecules are stabilized in the PVP host, which decreases their excitation energy (Paw and Eisenberg, 1997; Yaghi et al., 2003). It is still observed that the absorption band at 367 nm of Re-Gd@PVP fibers is enhanced obviously, compared to the MCLT absorption of Re-Gd (at 430 nm). This observation is attributed to the strong dipole moment of PVP which facilitates the MLCT transition of the Re-Gd dopant (Polo et al., 2006; Si et al., 2009).

The emission spectra of Re-Gd@PVP fibers (3 wt% and 5 wt%) are shown in Figure 3 as well. These emission bands peaking at 549 and 550 nm are rather similar to dopant Re-Gd and thus attributed to the emission from the MLCT excited state of Re(I)-based component. No emission from exciplex or Gd(III)-based component is observed. On the other hand, they (∼550 nm, FWHM = 78 nm) are blue-shifted and narrowed compared to the emission bands of dopant Re-Gd (556 nm in DMF solution, FWHM = 116 nm). This result suggests that the excited state in Re-Gd@PVP fibers [Re(I)-based component] has been immobilized and restricted by the PVP tight framework, so that the excited state is not free to lose and relax its energy, leading to the emission of blueshift and narrowed emission band.

**FIGURE 4:**
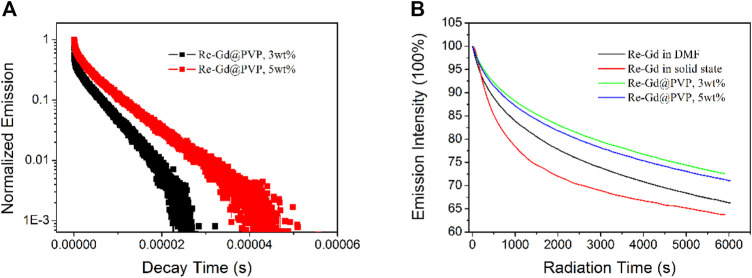
**(A)** Emission decay dynamics of Re-Gd@PVP. **(B)** Emission stability monitoring for Re-Gd in solid state and DMF solution, and Re-Gd@PVP.

The emission decay dynamics of Re-Gd@PVP fibers (3 wt% and 5 wt%) are shown in Figure 4. Biexponential decay pattern is observed, similar to the case of Re-Gd dopant. This observation suggests that dopant Re-Gd has preserved its MLCT-based emissive state in the PVP host. But their lifetimes are increased to 5.2 and 6.7 µs, compared to 4.9 µs (in solution) and 3.6 µs (in solid state) of Re-Gd dopant. These increased lifetimes are attributed to the immobilization effect from the PVP host which prevents the excited state from losing and relaxing its energy. Such polymer immobilization effect has been confirmed by other studies (Paw and Eisenberg, 1997; Yaghi et al., 2003). Yet, the biexponential decay pattern is still preserved and partially not desired, since there is still a short-lived emissive component in the excited state which was not efficient and applicable in producing ^1^O_2_, as mentioned previously. Considering the single emission band of Re-Gd and Re-Gd@PVP fibers (with no shoulder peaks or widened emission bands), these two emissive components shall come from one emissive state. The long-lived emissive component shall be attributed to the emissive decay of the MLCT excited state, while the short-lived one should be the dynamic process of the n-π* excited state (Si et al., 2009).

Given the aforementioned confirmed PVP immobilization effect, the Re-Gd molecules should be covered and protected by the PVP host, which shall decrease the photobleaching effect. To confirm this hypothesis, emission intensity monitoring on Re-Gd@PVP fibers (3 wt% and 5 wt%) is compared to that of Re-Gd dopant, as shown in Figure 4. Clearly, Re-Gd in a solid state suffers from an intense photobleaching effect. After 100 min of continuous excitation (*λ* = 350 nm), only 63% of the initial intensity is retrieved. Re-Gd in solution shows a better photostability by showing 66% of initial intensity after 100 min. This is because Re-Gd molecules are surrounded and isolated by solvent molecules so that Re-Gd molecules can transfer their excited energy to these solvent molecules, instead of decomposing Re-Gd molecular structure and thus leading to photobleaching. After being doped into PVP fibers, PVP offers a more rigid and tight environment for Re-Gd molecules, which explains the obviously improved photostability of Re-Gd@PVP fibers (72% retrieval for 3 wt% samples and 71% retrieval for 5 wt% sample). In addition, it is found that the photostability of low doping Re-Gd@PVP (3 wt%) is always better than that of high doping Re-Gd@PVP (5 wt%). This is explained by the concentration-induced aggregation. With the concentrated and aggregated Re-Gd molecules in PVP fibers, the probability of each Re-Gd molecule oxidized by surrounding neighboring excited Re-Gd molecules becomes higher, which leads to compromised photostability.

### ^1^O_2_-Producing Performance of Re-Gd@PVP Fibers

**FIGURE 5:**
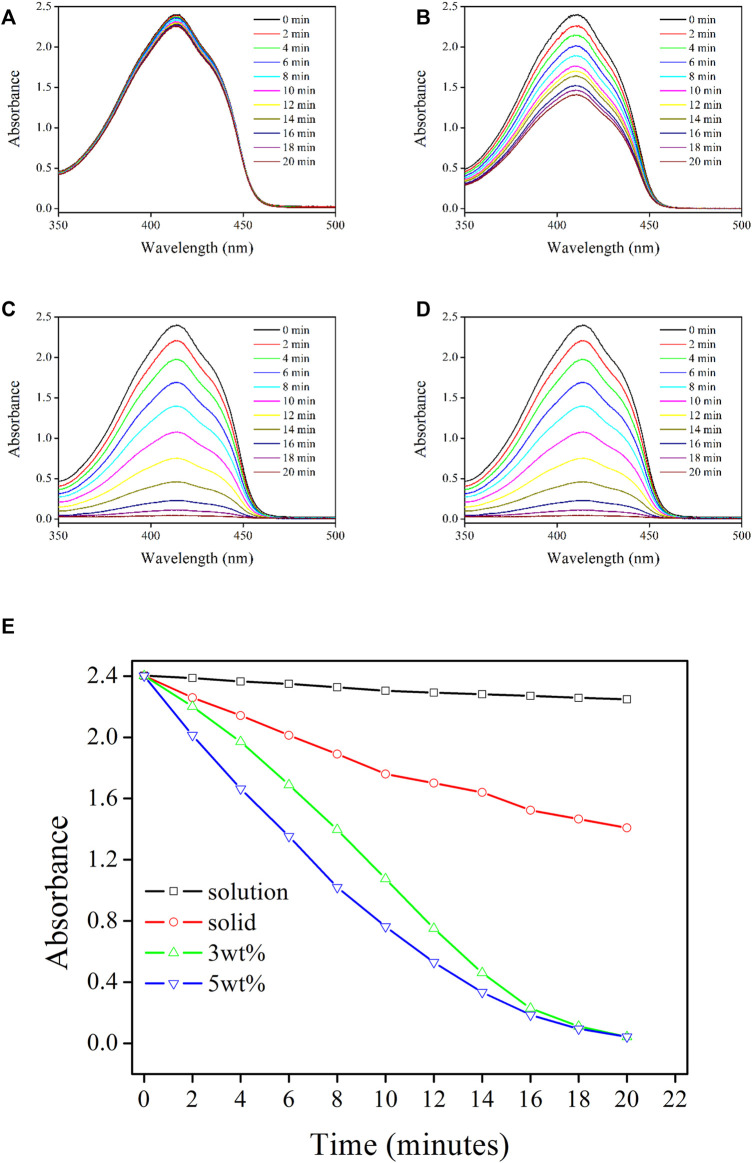
Absorption spectra of DPBF solution being treated by following samples: Re-Gd in solution **(A)** and solid state **(B)**, Re-Gd@PVP [3 wt%, **(C)** and Re-Gd@PVP (5 wt%, **(D)**], along with their absorbance variation monitoring **(E)**.

With the aforementioned confirmed positive factors for ^1^O_2_-producing in Re-Gd@PVP fibers, including dense but porous fibrous structure, long-lived excited state, and improved photostability, it is assumed that Re-Gd@PVP fibers shall be efficient in producing ^1^O_2_. Here DPBF is used as a ^1^O_2_ tracer whose absorption intensity at 410 nm is inevitably quenched by ^1^O_2_ and thus inversely proportionally to ^1^O_2_ concentration/quantity. As shown in Figure 5, DPBF absorption intensity is gradually and steadily quenched with an increasing amount of generated ^1^O_2_. In solution, the decreasing tendency is rather smooth. This is because the collision probability between the ^3^O_2_ stream (bubble) and photosensitizer is limited so the generated ^1^O_2_ amount is slim. In solid, a higher ^1^O_2_-producing efficiency is observed. On the other hand, the collision between the ^3^O_2_ stream and photosensitizer occurs only on the solid sample surface, which limits further improvement. There is still obvious DPBF absorption after 20 min of photoreaction. As for Re-Gd@PVP fibers, they have a much large surface area, so the collision probability between the ^3^O_2_ stream and photosensitizer is greatly improved. No obvious DPBF absorption is observed after 20 min of photoreaction, indicating a complete consumption of DPBF.

Although both Re-Gd@PVP samples (3 wt% and 5 wt%) have completely consumed DPBF after 20 min of photoreaction, it is observed that DBPF absorption is always quenched stronger by 5 wt% Re-Gd@PVP, compared to 3 wt% Re-Gd@PVP. This is because the absolute amount of photosensitizer (Re-Gd) in the former sample is higher than that in the latter sample, thus, given the comparable surface areas between two Re-Gd@PVP samples, the generated ^1^O_2_ amount from 5 wt% Re-Gd@PVP is higher than that from 3 wt% Re-Gd@PVP. For the last 4 min of photoreaction, nearly all DPBF tracers have been consumed, and the difference between ^1^O_2_-producing yields of Re-Gd@PVP samples becomes minor and neglectable. As a consequence, the highly efficient ^1^O_2_-producing from Re-Gd@PVP is confirmed.

### Further Confirmation of the Synergistic Effect *via* a Reference Photosensitizer

It can be found from the aforementioned discussion that there are two positive factors affecting Re-Gd@PVP ^1^O_2_-producing performance, which are the porous structure offered by electrospinning fibers and the improved ^1^O_2_-producing efficiency of photosensitizer (Re-Gd) through its synergistic effect between Re(I)-based component and Gd(III)-based component. To ultimately confirm the positive factor of synergistic effect on improving ^1^O_2_-producing efficiency, a reference photosensitizer (Re-NN) is applied and doped into PVP fiber *via* electrospinning (denoted as Re-NN@PVP) as well. Re-NN has exactly the same molecules of the Re(I) component of Re-Gd but has no Gd(III)-based component. The comparison between Re-Gd@PVP and Re-NN@PVP can give a direct confirmation of the synergistic effect. Following the same protocol, the ^1^O_2_-producing performance of Re-NN@PVP fibers (5wt%) is evaluated by the DPBF test. It is observed from Supplementary Figure S2 that although Re-NN@PVP gradually quenches DPBF absorption intensity, as Re-Gd@PVP does, it failed to completely consume all DPBF tracer at the end of photoreaction (20 min). An obvious DPBF absorption is observed by the end of photoreaction, indicating the absence of enough ^1^O_2_-producing efficiency.

For a detailed comparison, the excited state dynamics and photostability of Re-NN@PVP are determined and shown in Supplementary Figures S3, S4. It is observed from Supplementary Figure S3 that the excited state lifetime of Re-NN@PVP (∼6.7 µs) is comparable to those of Re-Gd@PVP (5.2 μs for 3 wt% and 6.7 μs for 5 wt%). The photostability of Re-NN@PVP is comparable to that of Re-Gd@PVP, as shown in Supplementary Figure S4. In this case, we finally attribute the improved ^1^O_2_-producing performance of Re-Gd@PVP, compared to Re-NN@PVP, to its Gd(III)-based component and synergistic effect. The positive effect of synergistic effect from dual-metal-core in photosensitizer Re-Gd is finally confirmed.

## Conclusion

In conclusion, we reported a dual-core metal photosensitizer Re-Gd with a synergistic effect which was proved to accelerate the transformation from ^3^O_2_ to ^1^O_2_. The photophysical parameters of Re-Gd and its fibrous films Re-Gd@PVP were carefully discussed and compared. It was found that the excited state lifetime and photostability of Re-Gd were both improved after being doped into fibrous films, favoring ^1^O_2_ generation. The ^1^O_2_ generation performance comparison between Re-Gd in the solid state, in solution, and fibrous films suggested that ^1^O_2_ generation performance was indeed improved by the electrospinning films. In addition, the positive factor of synergistic effect on improving ^1^O_2_-producing efficiency was confirmed by comparing Re-Gd@PVP films with reference films with a single-core metal photosensitizer having no synergistic effect.

There are points to be solved and improved though. First, the solubility of Re-Gd was poor which limited the doping concentration in PVP fibers. The fiber diameter was as large as ∼2 μm due to the high viscosity caused by Re-Gd. For further efforts, fiber diameter should be decreased to increase sample-specific area. Second, the photostability of the photosensitizer should be improved to ensure a stable and durable ^1^O_2_ generation. Finally, the lifetime of photosensitizer shall be further increased since there is still a short-lived emissive component in an excited state which was not efficient and applicable in producing ^1^O_2_. In other words, a long-lived single exponential decay pattern is desired for photosensitizers. Considering that there is a spin-orbit coupling effect in other rare-earth ions, they shall have the same function (synergistic effect). But, this hypothesis needs experiment evidence, so that the universality of this synergistic effect for similar singlet oxygen generating systems can be testified.

## Data Availability

The original contributions presented in the study are included in the article/Supplementary Material, further inquiries can be directed to the corresponding authors.
