# Supplementary material for: Improved Singlet Oxygen Production by Synergistic Effect via a Dual-Core Photosensitizer Doped Polymer Fibrous Films: Synthesis and Performance
Source: Front Chem. 2022 May 9;10:890545. doi: 10.3389/fchem.2022.890545 (PMC9124785; doi:10.3389/fchem.2022.890545)

Supplementary Material

# Supplementary Figures and Tables

Figure S1. Simulated geometry of Re-Gd, optimized by semi-empirical method PM6 with MOPAC2006. “Gd” was replaced by “La” during geometry optimization. Effective core potential (ECP) basis sets are usually used during calculation. In this case, it is hard to simulate the synergistic effect.


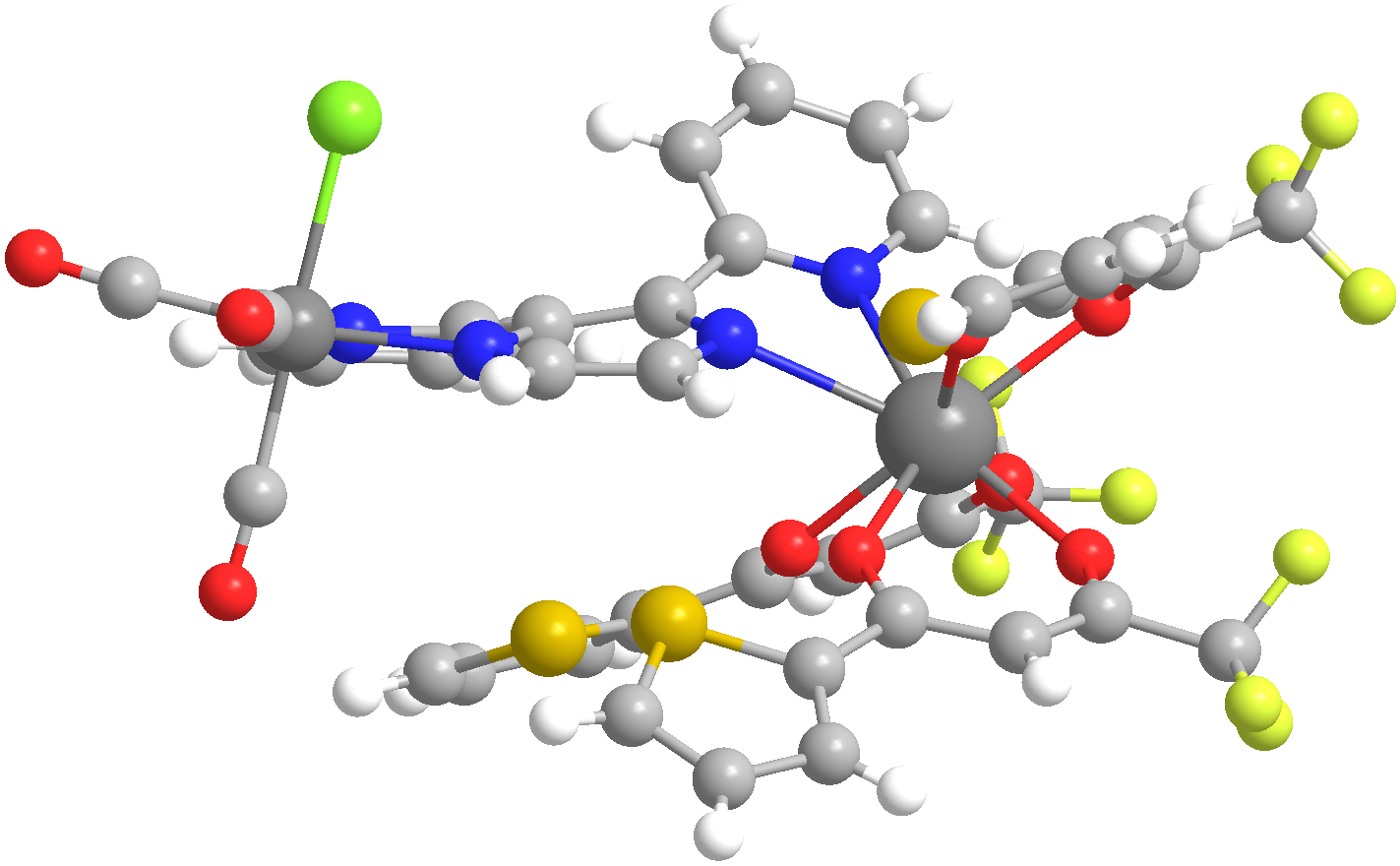


Below is detailed geometric parameters

CARTESIAN COORDINATES

NO. ATOM X Y Z

1 Gd 0.0000 0.0000 0.0000

2 Re 7.4016 0.0000 0.0000

3 Cl 7.1906 2.4525 0.0000

4 F -4.5529 2.2840 -0.4438

5 F -3.3747 3.5555 0.8141

6 F -3.7633 4.1358 -1.2188

7 F -2.5443 -0.1369 4.3097

8 F -0.7726 0.8851 4.9143

9 F -1.1022 -1.1494 5.5186

10 F -4.1035 -2.7060 -1.1653

11 F -4.1564 -0.8418 -2.1844

12 F -3.8042 -2.6322 -3.2767

13 O -1.6285 1.7033 0.0604

14 O 0.0497 1.0836 -2.0794

15 O -0.9893 -0.3142 2.1474

16 O 1.3679 -1.4965 1.1800

17 O -1.8277 -1.1593 -0.7931

18 O 0.7898 -1.5857 -1.5848

19 O 10.3745 0.3567 0.6479

20 O 7.6433 -3.0518 0.2309

21 O 8.1402 -0.1116 -2.9706

22 N 1.1396 1.6597 1.5896

23 N 2.5533 0.6512 -0.4564

24 N 5.2455 0.0885 -0.2380

25 N 6.6067 0.1623 2.0040

26 C 2.4842 1.8188 1.6182

27 C 3.1025 2.8212 2.3467

28 C 2.3392 3.6350 3.1452

29 C 0.9770 3.4242 3.2113

30 C 0.4222 2.4576 2.3949

31 C 3.2278 0.9525 0.6795

32 C 4.5500 0.5247 0.8489

33 C 4.6004 -0.0603 -1.3993

34 C 3.2324 0.1436 -1.4757

35 C 5.2724 0.3965 2.1309

36 C 4.6610 0.3646 3.3830

37 C 5.4253 0.1681 4.5157

38 C 6.7864 -0.0188 4.3853

39 C 7.3421 -0.0289 3.1232

40 C -2.2289 2.3200 -0.8726

41 C -3.4812 3.0809 -0.4244

42 C -1.9318 2.3596 -2.2059

43 C -0.8128 1.6938 -2.7717

44 C -0.6574 1.6741 -4.2274

45 C 0.1578 1.1596 -6.4797

46 C -1.0212 1.7889 -6.5027

47 C -1.5041 2.0903 -5.2239

48 C -0.4974 -0.7466 3.2288

49 C -1.2569 -0.3006 4.4976

50 C 0.6486 -1.4796 3.4339

51 C 1.5386 -1.8107 2.3952

52 C 2.7936 -2.5048 2.7400

53 C 5.0222 -3.4701 2.6451

54 C 4.6100 -3.3525 3.9076

55 C 3.3168 -2.8216 4.0785

56 C -2.0330 -1.8051 -1.8718

57 C -3.5217 -2.0177 -2.1338

58 C -1.1242 -2.3381 -2.7510

59 C 0.2768 -2.2653 -2.5075

60 C 1.1771 -3.0343 -3.3658

61 C 3.2152 -3.9399 -4.4112

62 C 2.1025 -4.5097 -4.8859

63 C 0.9277 -4.0035 -4.2967

64 C 9.2708 0.1862 0.3981

65 C 7.5114 -1.9243 0.1436

66 C 7.8867 -0.0610 -1.8523

67 S 0.7332 0.9045 -4.8865

68 S 3.8858 -2.9267 1.5182

69 S 2.8765 -2.7875 -3.2058

70 H 4.0422 2.9437 2.2955

71 H 2.7419 4.3343 3.6458

72 H 0.4341 3.9323 3.8041

73 H -0.5210 2.3510 2.4049

74 H 5.0846 -0.3085 -2.1785

75 H 2.7725 -0.0861 -2.2749

76 H 3.7195 0.4779 3.4577

77 H 5.0212 0.1606 5.3742

78 H 7.3337 -0.1372 5.1519

79 H 8.2754 -0.1772 3.0353

80 H -2.5034 2.8555 -2.7807

81 H 0.6233 0.8842 -7.2612

82 H -1.4812 2.0092 -7.3039

83 H -2.3299 2.5344 -5.0626

84 H 0.8477 -1.7754 4.3144

85 H 5.8690 -3.8222 2.4031

86 H 5.1525 -3.6093 4.6439

87 H 2.8642 -2.6914 4.9058

88 H -1.4380 -2.7659 -3.5392

89 H 4.0920 -4.1531 -4.7121

90 H 2.1038 -5.1872 -5.5513

91 H 0.0551 -4.3017 -4.5225

Figure S2. Left chart: Absorption spectra of DPBF solution being treated by Re-NN@PVP (5wt%). Right chart: its absorbance variation monitoring.


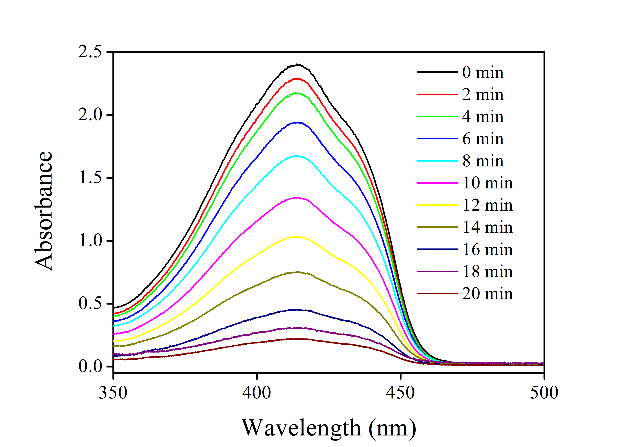

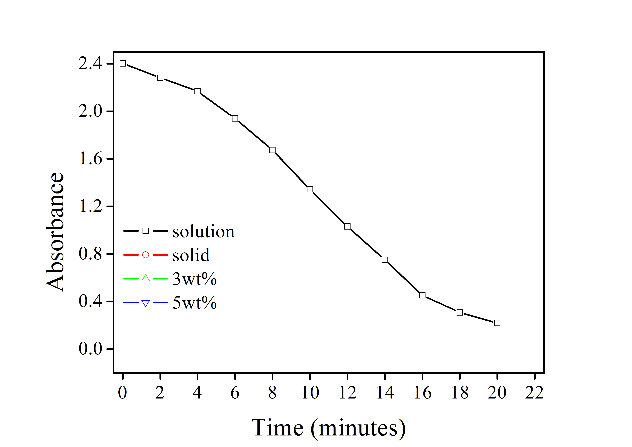


Figure S3. Excited state lifetime of Re-NN@PVP.


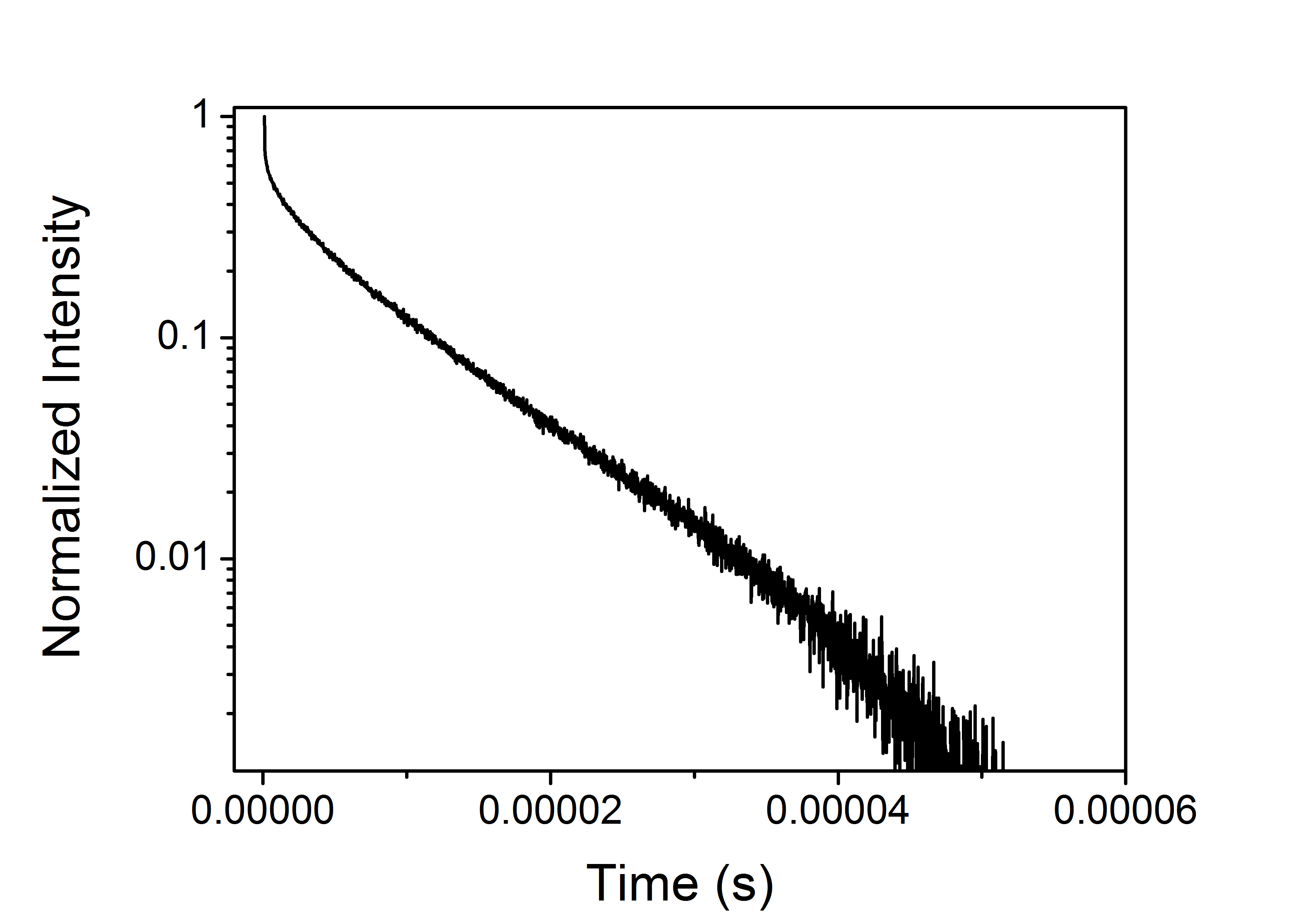


Figure S4. Emission intensity monitoring of Re-NN@PVP.


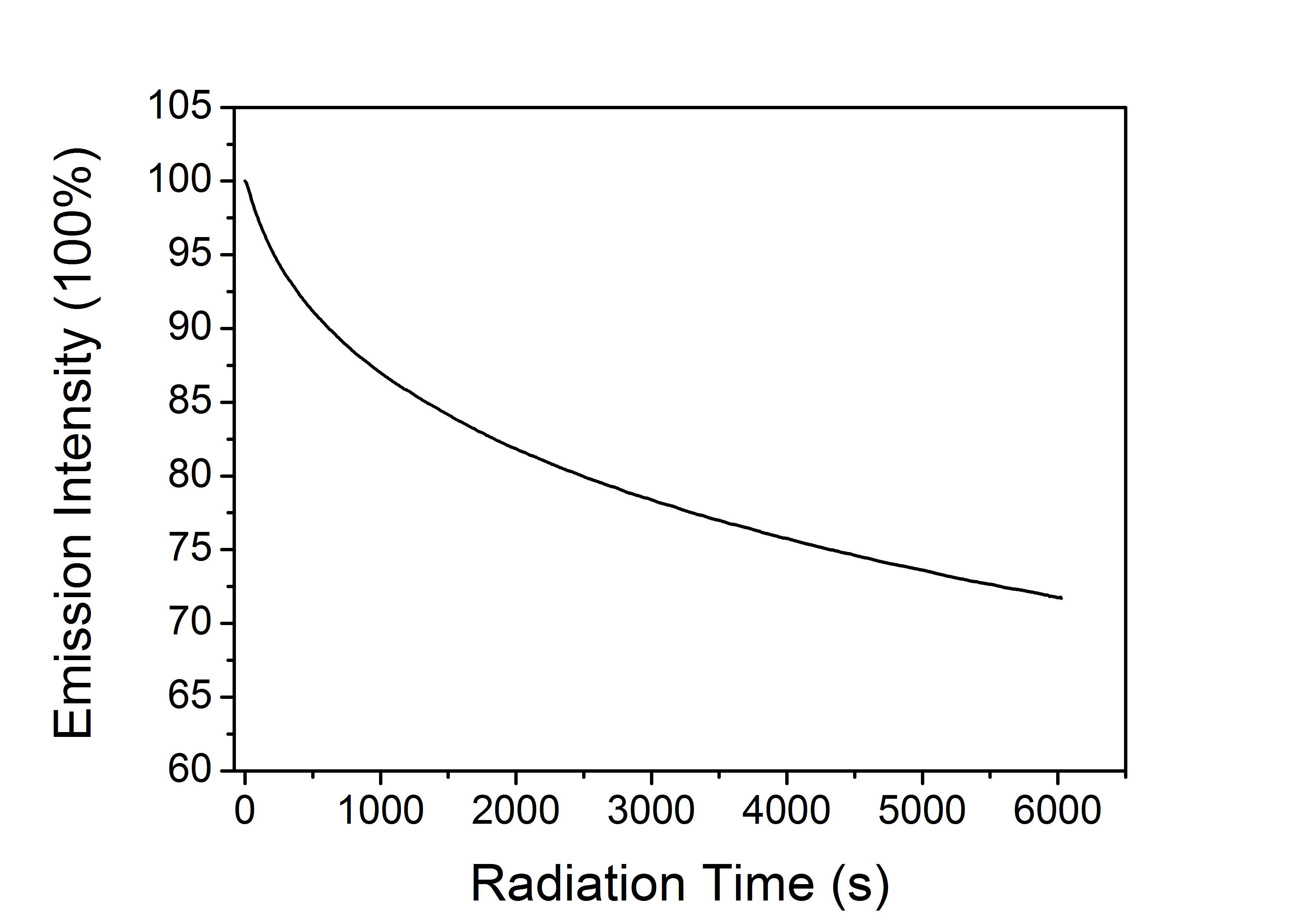

Supplement: Supplementary file 1 [file DataSheet1.docx]
